# EFFECTS OF CARBON VERSUS PLASTIC ANKLE FOOT ORTHOSES ON GAIT OUTCOMES AND ENERGY COST IN PATIENTS WITH CHRONIC STROKE

**DOI:** 10.2340/jrm.v56.35213

**Published:** 2024-08-23

**Authors:** Diana RIMAUD, Rodolphe TESTA, Guillaume Y. MILLET, Paul CALMELS

**Affiliations:** 1 Université Jean Monnet Saint-Etienne, CHU Saint-Etienne, Physical Medicine and Rehabilitation Department, Lyon 1, Université Savoie Mont-Blanc, Laboratoire Interuniversitaire de Biologie de la Motricité, Saint-Etienne; 2 Université Jean Monnet Saint-Etienne, CHU Saint-Etienne, Department of Orthopaedic Surgery, Lyon 1, Université Savoie Mont-Blanc, Laboratoire Interuniversitaire de Biologie de la Motricité, Saint-Etienne; 3 Université Jean Monnet Saint-Etienne, Lyon 1, Université Savoie Mont-Blanc, Laboratoire Interuniversitaire de Biologie de la Motricité, Saint-Etienne; 4 Institut Universitaire de France (IUF), Paris, France

**Keywords:** gait analysis, foot drop, ankle foot orthosis design, energy expenditure, hemiplegic patients

## Abstract

**Objective:**

To compare the walking performances of hemiplegic subjects with chronic stroke under 3 conditions: with a new standard carbon fibre ankle foot orthosis (C-AFO), with a personal custom-made plastic AFO (P-AFO), and without any orthosis (No-AFO).

**Design:**

Randomized, controlled crossover design.

**Patients:**

Fifteen chronic patients with stroke (3 women and 12 men, 59 [10] years, 13 [15] years since injury).

**Methods:**

Patients performed 3 randomized sessions (with C-AFO, P-AFO, no-AFO), consisting of a 6-min walk test (6MWT) with VO_2_ measurement and a clinical gait analysis. Energy cost (Cw), walking speed, spatio-temporal, kinetic, and kinematic variables were measured.

**Results:**

No significant differences were found between the C-AFO and P-AFO conditions. Distance and walking speed in the 6MWT increased by 12% and 10% (*p* < 0.001) and stride width decreased by -8.7% and -13% (*p* < 0.0001) with P-AFO and C-AFO compared with the No-AFO condition. Cw decreased by 15% (*p* < 0.002), stride length increased by 10% (*p* < 0.01), step length on affected leg increased by 8% (*p* < 0.01), step length on contralateral leg by 13% (*p* < 0.01), and swing time on the contralateral leg increased by 6% (*p* < 0.01) with both AFO compared with the No-AFO condition.

**Conclusion:**

The use of an off-the-shelf composite AFO (after a short habituation period) in patients with chronic stroke immediately improved energy cost and gait outcomes to the same extent as their usual custom-made AFO.

## LAY ABSTRACT

Gait impairments occur in more than 80% of stroke survivors and may impact walking speed and patients’ daily activities and autonomy. Ankle foot orthoses (AFOs) are often necessary to prevent foot drop, because of muscle weakness or muscle paralysis. Plastic AFOs are widely used in clinical practice. However, composite materials, especially carbon fibres, are now widely available for application in orthoses, although limited research has been conducted on their effects. The aim of this study was to explore the difference in walking performance in patients with stroke who have worn a new standard carbon fibre AFO or their personal custom-made plastic AFO. The use of the carbon AFO (despite a short habituation period) immediately improved energy cost and gait parameters such as distance, walking speed, stride, and step length, to the same extent as the typical custom-made AFO. This indicates improved stability and efficiency during walking, regardless of which AFO is used.

Stroke is the second leading cause of death and a major cause of long-term disability worldwide (1). Gait impairment occurs in more than 80% of stroke survivors (2), and the restoration of walking performance is usually the primary goal during rehabilitation (3). Between 70% and 80% of patients regain their ability to walk after 6 months (4) but, despite rehabilitation, 35% of all stroke survivors have residual gait impairments that require physical assistance (5). When walking ability is impaired, walking speed is significantly decreased (6) and a disrupted movement pattern increases energy cost (Cw) compared with healthy walking (7). The walking pattern (8) is altered due to muscle weakness, sensorimotor deficit, and spasticity, which impair the propulsion force during the stance phase, reduce toe clearance, and cause insufficient dorsiflexion during the swing phase due to foot drop. This reduced walking capacity has a great impact on patients’ daily life and autonomy, leading to sedentary behaviours, which in turn affects patients’ cardiovascular function and further limits their daily activities (9). Furthermore, these gait abnormalities place stroke survivors at high risk of falls (10).

Ankle foot orthoses (AFOs) are a common therapeutic choice for treating patients with impaired gait to prevent foot drop during the swing phase. AFOs improve the alignment of the ankle joint, provide mediolateral stability at the ankle in the stance phase, promote heel strike in the early stance, and facilitate toe clearance during the swing phase (11). An AFO allows patients to counteract their plantar flexor or dorsiflexor weakness, motor control deficits, spasticity, instability, and balance problems (11–14). All of these effects increase walking speed, improve balance, symmetry or mechanical work, and reduce Cw (15–19). Different categories of AFO exist, i.e., non-articulated, articulated, and posterior leaf spring (5). Historically, AFOs have often been manufactured as a custom-made rigid plastic piece, designed to partially immobilize the ankle joint at a neutral position (0 degrees) or in one or more planes. Plastic AFOs are routinely made of thermoplastics such as polypropylene. This material is widely used in clinical practice due to its many advantages, such as its relatively low cost, good aesthetics, and ease of cleaning (20).

Composite materials, including carbon fibres, are now widely available for application in orthoses. A composite AFO is able to significantly improve pathological walking by storing energy during deformation and increasing push-off during the pre-swing. Various studies have shown that a composite AFO can decrease the energy expenditure of disabled patients (21, 22). However, limited research has been conducted on composite AFOs compared with plastic manufactured AFOs which are usually prescribed.

In this context, the objective of this study was to compare the effects of an “off the shelf” composite AFO (C-AFO) with those of a custom-made plastic AFO (P-AFO) and with a control condition without an orthosis (shoes only: No-AFO) on walking ability and Cw during walking in post-stroke hemiplegic patients. Our secondary objectives were to investigate the kinetic and kinematic variables according to these 3 conditions.

## METHODS

### Study design and ethics

This randomized controlled crossover study was conducted in accordance with the Consolidated Standards of Reporting Trials (CONSORT) recommendations and the intervention was reported based on the template for the intervention description and replication (TIDieR) checklist and guide. The protocol was performed according to the Declaration of Helsinki and was approved by the ethics committee (Comité de Protection des Personnes Ile de France VII, #20-003). The study was registered with the International Registry of Clinical Trials (www.clinicaltrials.gov; ID: NCT04323943; trial registry name: Mecaspry). Prior to participation in the study, all participants were informed verbally and in writing about the objectives, risks, and benefits of the intervention. All patients signed an informed consent form.

This was a non-blinded study but, to reduce bias, all of the data analysis was performed by the same evaluator, who was blinded to patient group allocation.

### Participants

This study was proposed to all consecutive patients who were treated in the Physical Medicine and Rehabilitation Unit of the Saint-Etienne University Hospital and met the following inclusion criteria: (*i*) had hemiplegia following stroke for more than 6 months, (*ii*) were over 18 years of age, (*iii*) were able to walk at least 15 m barefoot, and (*iv*) had used a custom-made thermoplastic AFO for at least 3 months. During a medical consultation, the principal investigator proposed that the eligible patients participate in this protocol, gave them a precise description of the study and the patient information leaflet, and allowed them time to reflect. When a subject agreed to participate, he/she was scheduled for an inclusion visit to the Clinical and Exercise Physiology Department of the Saint Etienne University Hospital.

### Experimental protocol

**Fig. 1:**
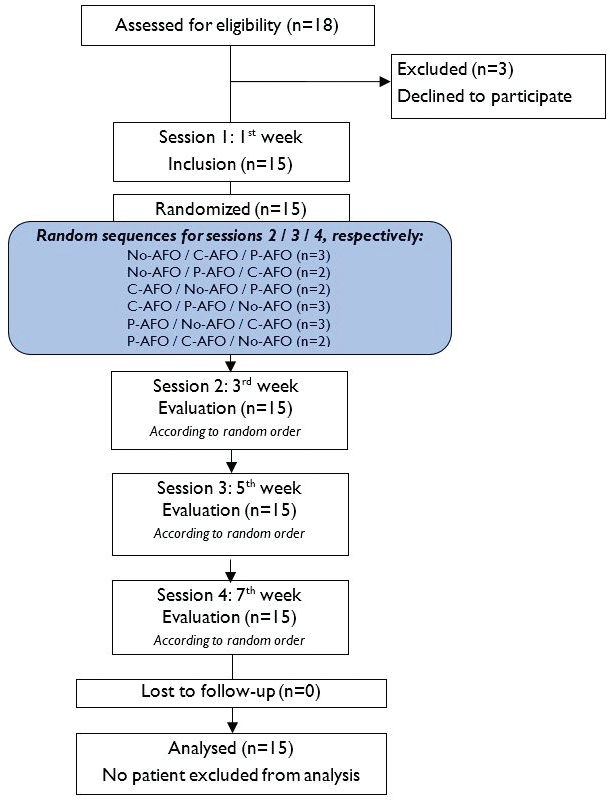
Study flowchart.

Each enrolled participant completed 4 sessions, with 2 weeks (± 2 days) between the sessions (see flowchart in Fig. 1). The total study duration for a patient was approximately 6 weeks. After screening for inclusion and exclusion criteria, the first visit (inclusion visit) consisted of a clinical evaluation of leg motor function (Fugl-Meyer scale [23]), spasticity (Modified Ashworth scale [24]), and passive range of motion (hip, knee, ankle). Individuals with symptomatic hyperextension (hyperextension of the knee beyond 5°) or a flexed knee (i.e., an attitude of the knee joint that cannot extend fully) were also noted.

The patients were then familiarized with the different walking tests with all of the equipment to avoid learning effects. Verification of the quality of the P-AFO and installation/adjustment/testing of the C-AFO were performed.

The next 3 visits included the same evaluations with the 3 investigated conditions: with P-AFO, with C-AFO, and with No-AFO, in random order.

Simple randomization of the order of interventions was conducted by a study coordinator who was not involved in the assessment or data analysis processes, and allocation was performed at the first visit (6 x 3 crossover design, random sequences in Fig. 1).

Two weeks prior to the C-AFO evaluations, the subject was asked to wear and walk with the C-AFO. The same applied to the P-AFO evaluation. In the 2 weeks prior to the No-AFO evaluation, the subjects were allowed to wear and walk with their preferred orthosis.

### Ankle foot orthosis

**Fig. 2:**
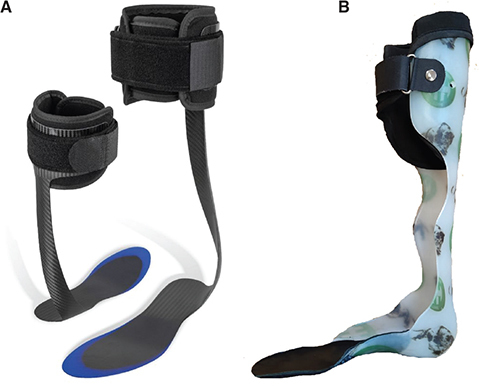
(A) Composite ankle foot orthosis (SpryStep^®^ Original, Thuasne, France) and (B) an example of a custom-made thermoplastic ankle foot orthosis.

*Composite ankle foot orthosis (C-AFO).* Each subject was fitted with an off-the-shelf composite AFO (SpryStep^®^ Original, Thuasne, France) based on a sizing table (Fig. 2A). This AFO is composed of 2 parts (rigid and soft parts) that are already assembled. The blue translucent area on the footplate was trimmable. A physical medicine and rehabilitation doctor performed the initial fitting and adjustments.

*Custom-made plastic ankle foot orthosis (P-AFO).* The custom thermoplastic AFOs were all performed by the same CPO (Certified Prosthetist/Orthotist), and were all custom made for each patient at least 3 months previously due to an indication of post-stroke foot drop (Fig. 2B). No dynamic articular system on the ankle or foot was used.

For each gait analysis, the subjects wore their usual shoes. The use of a walking aid was identified and maintained during testing when necessary.

### Evaluations

*Installation of equipment.* Before the tests were performed, each patient was fitted with sensors (for gait analysis) and a mask from the portable Metamax system (Metamax 3B, 264 Cortex Biophysik, Leipzig, Germany) to record oxygen consumption. This allowed all of the tests and measurements to be performed using the equipment already in place.

*Six-min walk test.* After 15 min of rest, the 6MWT was conducted according to the American Thoracic Society/European Respiratory Society (ATS/ERS) guidelines (25). The 6MWT is a self-paced test of walking capacity. Patients were asked to walk as far as possible in 6 min along a 30-m flat corridor. The distance in metres was recorded. Standardized instructions and encouragement were given during the test (25).

*Gait analysis during a 5×15-m walk.* At the end of the 6MWT, the patient walked immediately and without interruption to the gait analysis platform (at the end of the corridor) to perform a 5×15-m walk at a comfortable walking speed.

### Outcomes and measurements

*Walking performance.* The primary outcome was the distance covered in the 6MWT. Walking speed (in m·s^-1^), number of steps, and cadence (counted in steps per min, a task performed by a dedicated experimenter using an electronic finger counter) were also calculated from the 6MWT. At the end of the 6MWT, the perceived exertion (RPE) rating was recorded with the Perceived Exertion Scale (26).

To analyse the characteristics of the patients whose walking speed improved with the C-AFO, we performed a 2-subgroup analysis: a group of patients who responded to the composite orthosis (i.e., “responding group”: patients for whom the C-AFO improved their gait speed compared with the P-AFO), and a “non-responding group” (patients for whom the C-AFO did not improve their walking speed compared with the P-AFO).

*Energy cost of walking.* Heart rate and breath-by-breath oxygen uptake (V̇O_2_) were measured at rest for 10 min before the 6MWT, and continuously during the 6MWT, using the Metamax portable system. The energy cost (Cw, in ml O_2_·kg^-1^.m^-1^) was calculated as VO_2_ (i.e., net oxygen consumption during walking) divided by the walking speed, as follows (22, 27):


$$Cw=\frac{VO_{2}-VO_{2}\;at\;rest}{walking\;speed}$$

*Gait analysis variables.* Gait was analysed during the 5×15-m walk using a motion capture system with 12 optoelectronic cameras (Motion Analysis Corporation, Santa Rosa, CA, USA). The trajectories of 25 reflective markers were acquired. Markers were placed on anatomical landmarks in respect of the biomechanical model developed at Instituto Orthopedico Rizzoli (IOR) (28). As all of the tests were performed with shoes, the foot markers were placed directly on the shoes on the first and fifth metatarsal heads and on the calcaneus. Trajectories were recorded and reconstructed using Cortex 7 software (Motion Analysis Corporation, Santa Rosa, CA, USA).

The ground reaction forces were acquired synchronously with the kinematic data using 8 force plates (Model 9287C, Kistler, Winterthur, Switzerland) staggered along the walkway. Once the data were collected, we used Visual3D Software (C-Motion, Germantown, MD, USA) to reconstruct the joint kinematics and moments following the recommendations of the International Society of Biomechanics (29). Spatiotemporal variables, joint kinematics, joint moments, and ground reaction forces were calculated using Visual3D software.

The gait cycle was defined as the time interval between 2 successive foot strikes on the same side. During the gait sessions, 3 types of variables were calculated: spatiotemporal, kinematic, and kinetic gait variables.

The following spatiotemporal variables of the gait cycle (GC were quantified: walking speed m·s^-1^) cadence (no. of steps/min), stride length (m), stride width (m), cycle time (s), and double support time (s). Spatiotemporal variables of the gait cycle on steps were quantified for both limbs (paretic and non-paretic): cadence (steps/min), step length (m), step time (s), stance time % of GC), and swing time (% of GC). Foot strike and foot-off events were detected from data acquired with the force plates.

Kinematic variables were quantified for both limbs (paretic and non-paretic): maximal angle values (degrees) of knee and hip flexion and extension, ankle plantar flexion and dorsiflexion, min/max foot progression and min/max pelvic obliquity.

Kinetic variables for both limbs (paretic and non-paretic) were normalized for body weight and reported in watts per kilogram (W/kg) for peak ankle power, peak knee power, peak hip power, and antero-posterior ground reaction force (N/kg).

*Satisfaction questionnaire.* Patient satisfaction with both AFOs was also evaluated at the end of the study using a specific 7-item questionnaire (“What effects did you feel when you walked? For each item, please indicate the number from 0 to 10 that most closely corresponds to your situation on the visual analogue scale (VAS), considering: 0 = Not at all, 10 = Absolutely”). A question on overall comfort when walking and a question on their preferred AFO were also asked.

### Statistical analysis

The mean and standard deviation (SD) were calculated for all the measured variables. The normality of the distributions of all of the values was verified with the Kolmogorov–Smirnov test, and the homogeneity of the variances was verified with Fisher’s F test. As the conditions of normality and homogeneity of variances being fulfilled for each variable, repeated measures ANOVA (C-AFO, P-AFO, and No-AFO) was applied for each variable (6MWT, kinematic, and kinetic variables). If a significant effect was found using repeated-measures ANOVA, post hoc analyses with Bonferroni correction for pairwise comparisons were applied.

To compare satisfaction scores between the use of C-AFO and P-AFO, a Wilcoxon test was used because the conditions of normality were not met for this variable.

To compare quantitative data between the responding and non-responding groups, a *t*-test was used. A χ^2^ test was used to compare qualitative data (i.e., sex, presence of hyperextension or flexed knee, affected leg, mobility aids) between these 2 groups. The significance level was set to *p* < 0.05. Statistical analysis was performed using IBM SPSS Statistics for Windows, version 21.0 (IBM Corp., Armonk, NY, USA).

The sample size was calculated using RStudio software 1.3.959 (RStudio, PBC, Boston, MA, USA) and estimated from a previous study showing a difference in walking speed between No-AFO and C-AFO conditions of 0.07 (0.04) m/s, in 10 participants with chronic stroke (27). The sample size required was 12, based on a power of 80% and a 2-tailed alpha level of 0.05. Considering a dropout rate of 20%, 15 subjects were required to detect a significant difference in walking speed.

## RESULTS

Eighteen patients were considered to be potentially eligible for the study between September 2020 and September 2021. Of these, 15 were included (3 patients declined to participate) and completed the entire study. In summary, the patients had a mean age of 59.0 (SD 10.8) years (12 men and 3 women), presented with chronic stroke (mean time since stroke 13.3 [SD 15.0] years), had been using a custom-made thermoplastic AFO for a mean of 5.9 (SD 4.3) years (min: 5 months and max: 13 years). The patients’ characteristics are detailed in Table I.

**Table I:** Demographic and disease-related characteristics of the participants

Characteristics	*n*	Mean (SD)
Age (years)		59.0 (10.8)
Time since stroke (years)		13.3 (15.0)
Height (m)		1.74 (0.07)
Weight (kg)		78.3 (10.5)
Body mass index (kg/m²)		25.7 (2.8)
Sex		
Men	12	
Women	3	
Affected leg		
Left	10	
Right	5	
Stroke type		
Ischaemic	7	
Haemorrhagic	8	
Mobility aids		
None	8	
Cane	7	
Fugl-Meyer assessment (scores)		
Total motor score (0–100)		50.3 (22.4)
Upper extremity subscore (0–66)		29.2 (20.3)
Lower extremity subscore (0–34)		21.1 (4.3)
Ashworth scale scores (0–4)		
Hip flexors		0
Knee flexors		1
Ankle dorsiflexors		1
ROM		
Ankle		
Knee		
Hip		
MRC score (0–3)		
Hip flexors (0–3)		2
Hip extensors (0–3)		2
Knee flexors (0–3)		2
Knee extensors (0–3)		2
Ankle dorsiflexors (0–3)		1
Ankle plantar flexors (0–3)		1

SD: standard deviation; MRC: Medical Research Council.

### Walking performance and energy cost of walking

Table II indicates the walking performance and energy cost in the 6MWT. No significant differences were found between the C-AFO and P-AFO conditions.

**Table II:** Comparison of walking performance and energy cost during the 6MWT under the 3 conditions

Factor	6MWT		
	No-AFO Mean (SD)	P-AFO Mean (SD)	C-AFO Mean (SD)
6MWT (m)	**269.0**^**a**^ **(155.8)**	**301.9**^**b**^ **(148.9)**	**296.8**^**b**^ **(160.1)**
Walking speed (m·s^-1^) (i.e., at maximal speed)	**0.75**^**a**^ **(0.43)**	**0.84**^**b**^ **(0.41)**	**0.82**^**b**^ **(0.44)**
Steps (number)	558.8 (177.8)	573.8 (163.0)	564.7 (174.0)
VO_2_ rest (ml.min.kg^-1^)	3.6 (0.6)	3.5 (0.7)	3.7 (1.1)
VO_2_ peak (ml.min.kg^-1^)	15.7 (5.1)	15.3 (4.8)	15.3 (5.2)
VO_2_ 6MWT (ml.min.kg^-1^)	13.4 (4.1)	13.4 (4.2)	13.2 (4.3)
Energy cost (mlO_2_.kg^-1^m^-1^)	**0.27**^**a**^ **(0.15)**	**0.23**^**b**^ **(0.13)**	**0.23**^**b**^ **(0.12)**

The same superscript letters indicate no significant difference in the same variable between the 3 different conditions. Significant *p*-values are represented in bold.

SD: standard deviation; 6MWT: 6-min walk test; VO_2:_ oxygen uptake; RER: respiratory exchange ratio; HR: heart rate; RPE: rating of perceived exertion; No-AFO: shoes only; P-AFO: custom-made plastic AFO; C-AFO: composite AFO.

The distance walked during the 6MWT was significantly longer with the P-AFO (301.9 [SD 148.9] m, +12%, *p* = 0.013) and the C-AFO (296.8 [SD 160.1] m, +10%, *p* = 0.005) compared with walking without an AFO (269 [SD 155.8] m). Similarly, the average walking speed during the 6MWT (i.e., at maximal speed) was significantly faster with both AFOs (+12% with the P-AFO, *p* = 0.013; and +10% with the C-AFO, *p* = 0.005) than under the No-AFO control condition.

Cw was significantly 15% higher with No-AFO (0.27 [SD 0.15] mlO_2_·kg^-1^m^-1^) than with the P-AFO (0.23 [SD 0.13] mlO_2_·kg^-1^m^-1^, *p* = 0.024) and the C-AFO (0.23 [SD 0.12] mlO_2_·kg^-1^m^-1^, *p* = 0.024).

### Spatiotemporal variables

The spatiotemporal variables obtained during the 5×15-m walk test are given in Tables III and IV. No significant differences were found between the C-AFO and P-AFO tests for any of the spatiotemporal variables.

**Table III:** Spatiotemporal variables during the 5×15-metre walk under the 3 conditions

Factor	No-AFO Mean (SD)	P-AFO Mean (SD)	C-AFO Mean (SD)
Walking speed (m·s^-1^) (i.e., at comfortable speed)	0.67 (0.38)	0.74 (0.37)	0.74 (0.38)
Stride length (m)	**0.88**^**a**^ **(0.33)**	**0.96**^**b**^ **(0.33)**	**0.96**^**b**^ **(0.33)**
Stride width (m)	**0.23**^**a**^ **(0.04)**	**0.21**^**b**^ **(0.04)**	**0.20**^**b**^ **(0.03)**
Cycle time (s)	1.50 (0.71)	1.52 (0.64)	1.53 (0.63)
Double support time (s)	0.74 (0.54)	0.68 (0.52)	0.69 (0.52)

The same superscript letters indicate no significant difference in the same variable between the 3 different conditions. Significant *p*-values are represented in bold.

SD: standard deviation; No-AFO: shoes only; P-AFO: custom-made plastic AFO; C-AFO: composite AFO.

**Table IV:** Spatiotemporal variables during the 5×15-m walk on steps of paretic and non-paretic legs under the 3 conditions

	Paretic leg			Non-paretic leg		
	No-AFO Mean (SD)	P-AFO Mean (SD)	C-AFO Mean (SD)	No-AFO Mean (SD)	P-AFO Mean (SD)	C-AFO Mean (SD)
Step length (m)	**0.49**^**a**^ **(0.15)**	**0.53**^**b**^ **(0.13)**	**0.53**^**b**^ **(0.13)**	**0.38**^**a**^ **(0.19)**	**0.43**^**b**^ **(0.20)**	**0.43**^**b**^ **(0.20)**
Step time (s)	0.93 (0.42)	0.90 (0.41)	0.88 (0.38)	0.63 (0.26)	0.63 (0.23)	0.65 (0.26)
Step phase in % GC	**60%**^**a**^ **(4)**	**58%**^**b**^ **(4)**	**57%**^**b**^ **(4)**	**40%**^**a**^ **(4)**	**42%**^**b**^ **(4)**	**43%**^**b**^ **(4)**
Stance phase in % GC	67% (7)	66% (6)	66% (7)	**78%**^**a**^ **(6)**	**76%**^**b**^ **(7)**	**75%**^**b**^ **(7)**
Swing phase in % GC	33% (7)	34% (6)	34% (6)	**22%**^**a**^ **(7)**	**24%**^**b**^ **(6)**	**25%**^**b**^ **(7)**
Cadence (steps/min)	73.5 (23.1)	75.6 (21.8)	77.0 (23.0)	106.6 (27.6)	104.0 (24.5)	102.7 (27.5)

The same superscript letters indicate no significant difference in the same variable between the different conditions. Significant *p*-values are represented in bold.

SD: standard deviation; No-AFO: shoes only; P-AFO: custom-made plastic AFO; C-AFO: composite AFO; GC: gait cycle.

The comfortable walking pace at speed was faster with both AFOs (0.74 [SD 0.37] m·s^-1^, +9% with the P-AFO; 0.74 [SD 0.38] m·s^-1^, +10% with the C-AFO) than in the No-AFO condition (0.67 [SD 0.38] m·s^-1^, *p* = 0.045, but not significant after Bonferroni correction) (Table III).

Stride length was significantly improved with both AFOs, i.e., 0.96 (SD 0.33) m, +9% with both AFOs compared with the No-AFO condition (0.88 [SD 0.33] m, *p* < 0.001). The stride widths were 0.21 (SD 0.04) m, -9% with the P-AFO (*p* = 0.003) and 0.20 [SD 0.03] m, -13% with the C-AFO (*p* < 0.001), compared with the No-AFO condition (0.23 [SD 0.04] m).

The step length for both the paretic and non-paretic legs was significantly enhanced with both AFOs as opposed to the No-AFO condition (0.53 [SD 0.13] m, +8% for the paretic leg with both AFOs, *p* = 0.005); and +13% (0.43 [SD 0.20] m for the non-paretic leg with both AFOs, *p* = 0.002) (Table IV).

Compared with that of No-AFO condition, the step phase on the non-paretic leg (in % of GC) increased by +3% with the C-AFO (*p* < 0.001) and +2% with the P-AFO (*p* = 0.03); and the step phase of the paretic leg decreased by -3% with the C-AFO (*p* < 0.001) and -2% with the P-AFO (*p* = 0.04).

For the non-paretic leg, the swing phase increased significantly from 22% (with No-AFO) to 25% with the C-AFO (*p* = 0.02) and to 24% with the P-AFO (*p* = 0.02), and the stance phase decreased significantly from 78% (with No-AFO) to 75% with the C-AFO (*p* < 0.001) and to 76% with the P-AFO (*p* = 0.001). There were no significant differences between the conditions for cycle time, double support time, or cadence.

### Kinematic and kinetic variables

No significant differences were found between the C-AFO and P-AFO tests in terms of kinematic variables.

As expected for AFOs, the ankle plantar flexion angle of the paretic limb decreased significantly by 40.6% (-6.5°) with the P-AFO and by 43.1% (-6.9°) with the C-AFO (*p* < 0.0001) compared with that in the condition without orthoses (Table SI).

In the non-paretic leg, there was a decrease in the maximal knee extension angle (knee hyperextension) measured during the stance phase with both AFOs, compared with the No-AFO condition (*p* = 0.0247).

The kinetic variables (Table SII) did not differ among the 3 conditions.

*Effects of composite ankle foot orthoses.* Eight patients showed improved walking speed with the C-AFO compared with their P-AFO (+4% [SD 11]) and they composed the responding group. Seven patients showed decreased walking speed with the C-AFO (-11% [SD 11]) and they composed the non-responding group.

Our results showed that patients with knee hyperextension were significantly more common (*p* = 0.003) in the responding group (6 patients with symptomatic hyperextension) than in the non-responding group (no patients with symptomatic hyperextension).

Conversely, patients with a flexed knee were significantly more common (*p* = 0.012) in the non-responding group (4 patients with a flexed knee) than in the responding group (no patient with a flexed knee).

### Satisfaction questionnaire

Satisfaction scores were significantly higher with C-AFO than with P-AFO for the items “I take bigger steps” (*p* = 0.03) and “I have less fatigue” (*p* = 0.02) (Table V). Some 60% of patients reported that of the 2 AFOs, the C-AFO was the orthosis with which they felt most comfortable when walking, and 67% of patients wished to keep the C-AFO at the end of the study.

**Table V:** Satisfaction with P-AFO vs C-AFO

	VAS	
	P-AFO Mean (SD)	C-AFO Mean (SD)
I have less foot drop	6.4 (2.6)	7.4 (2.4)
I have less spasticity	4.1 (3.0)	5.0 (4.1)
My foot catches the ground less	6.5 (2.4)	7.4 (3.0)
Less leg circumduction	5.0 (3.7)	6.5 (3.7)
**I take bigger steps**	6.5* (2.4)	7.1* (3.4)
**I have less fatigue**	5.1* (3.0)	8.0* (1.9)
I have less pain	4.0 (3.3)	7.3 (3.0)

Significant *p*-values are represented in bold.

* *p* < 0.05: indicate significant difference in the same variable between the two conditions. VAS: Visual Analogue Scale (0 = “not at all”; 10 = “absolutely”); SD: standard deviation; No-AFO: shoes only; P-AFO: custom-made plastic AFO; C-AFO: composite AFO.

## DISCUSSION

The primary results of the present study indicate that using an off-the-shelf composite AFO significantly improved walking performance (i.e., walking speed and distance during the 6MWT) and energy cost, to the same degree as their custom-made thermoplastic AFO compared with a control condition without AFO. It was also found that AFO use improved other walking variables, such as decreasing the plantar flexion ankle angle of the paretic limb, combined with an improved stride width and non-paretic swing time. Interestingly, no differences between the results for the 2 types of AFO were observed, despite their very different designs. However, patients with knee hyperextension showed improved walking speed with the C-AFO compared with their P-AFO, while patients with a flexed-knee gait did not.

### Walking performance and energy cost of walking

The benefits of AFO use on walking performance in our study are consistent with a recent meta-analysis by Choo and Chang (30). Regardless of the AFO used, they also observed significant improvements in walking speed, step length, and stride length.

An immediate 15% reduction in energy cost was found when wearing a composite or thermoplastic AFO while walking quickly. The fact that a thermoplastic or composite AFO decreases the energy cost in subjects with stroke during walking has already been observed in many studies (19, 22, 27, 31), but to our knowledge no studies have directly compared a P-AFO with a C-AFO.

Our results are consistent with those of Danielsson & Sunnerhagen (27), who showed that the use of a composite AFO in patients with stroke may decrease energy cost by 12% during 5 min of walking at a comfortable speed. More recently, Daryabor et al. (19) conducted a systematic review on the efficacy of AFO, particularly on energy expenditure during walking after chronic or subacute poststroke hemiplegia. Considering the data of 15 studies involving 195 participants, with most patients in the chronic phase, they found that AFOs have immediate positive effects on energy cost and the physiological cost index. As suggested by this systematic review, the reduction in Cw with an AFO during walking may stem from increased speed and balance during gait (16, 18). This also seems to be the case in our study. Indeed, a reduction in stride width during walking with an AFO indicates the patient’s capacity to walk with a reduced base of support. If patients are able to walk faster while reducing the base of support, this means that they have improved their dynamic balance and that their postural adjustment strategy is better with the AFO. Similarly, if patients are able to walk faster without increasing their energy expenditure (in our study, VO_2_ did not change during walking even if they walked faster), the energy cost is reduced.

In addition, in healthy subjects, as walking speed increases or decreases from their preferred walking speed, walking becomes less economical; therefore, the energy economy curve is U shaped (32). In subjects with chronic poststroke hemiparesis, some studies have shown that the preferred walking speed is significantly slower and paradoxically is not the most economical. Indeed, by walking faster, these patients would require less energy per metre travelled (33). In a recent study, Awad et al. (34) showed that faster walking speeds were 9% more economical than slower walking speed in 7 individuals with chronic hemiparesis, and that individuals with slower walking speeds had an enhanced energetic benefit when walking faster. Therefore, we can hypothesize that if patients walk faster with an AFO (due to a possible improvement in the push-off phase), then an increase in walking speed would result in a decrease in the energy cost of walking.

It could also be argued that the reduction in Cw with an AFO could also occur due to greater coordination and biomechanical effectiveness with an AFO. We tried to record muscle activity in the lower limbs using electromyography, but unfortunately the wireless captors did not fit under the AFOs when walking, so the signals could not be analysed for the entire population with a sufficient guarantee of quality. We were therefore not able to verify whether the reduction in Cw when wearing an AFO occurred, at least in part, due to reduced spasticity or co-contraction of some leg muscles. However, this analysis seems essential to consider, especially in patients in the post-acute phase and during post-stroke neuromotor recovery. In this population of chronic patients, it is perhaps more difficult to modify motor activity patterns immediately.

### Kinematic, kinetic, and spatio-temporal variables

Regarding the spatiotemporal variables, the increase in stride and step length and the decrease in stride width and non-paretic swing time with both AFOs suggests an improvement in dynamic balance abilities. These results are consistent with the literature, showing the beneficial effect of wearing an AFO on these variables (16, 17, 35), and are in concordance with our other results showing better temporal symmetry, as the distribution of step time on the paretic leg versus the non-paretic leg improved from 60% vs 40% without an AFO, to 58% vs 42% (with the P-AFO) or 57% vs 43% (with the C-AFO).

Our results also showed that there was a significant decrease in the maximal knee extension angle on the non-paretic leg during the stance phase when wearing an AFO. This suggests that the significant decrease in ankle plantar flexion due to the AFO on the paretic limb facilitates stepping of the paretic leg during the swing phase and, consequently, limits hyperextension of the non-paretic leg. This can be considered an interesting result and needs to be confirmed and analysed in a more specific population, as it may be related to motor control of the knee, the existence of co-contraction, or spasticity. Therefore, such changes when using an AFO lead to a more normalized gait, with greater stability during walking, better control of ankle plantar flexion and dorsiflexion, and improved walking speed and Cw.

### Comparison between C-AFO and P-AFO

The present study is the first to compare the effects of an off-the-shelf composite AFO with a plastic custom-made AFO on a very large variety of variables, such as Cw, spatiotemporal, kinetic, and kinematic analysis in patients with stroke. Our results did not show that one AFO is more effective than the other in our sample population. This finding is consistent with the few studies that have investigated the effects of different AFOs on Cw but that reported no significant difference between AFOs (36, 37). Studies comparing different types of AFO on other parameters are limited and under-sampled, leading to inconclusive results (18, 19, 40), and the quality of evidence in favour of 1 type of AFO compared with others seems to be low to very low (18, 40). However, even if the C-AFO did not show superior efficacy, our results demonstrated that we can obtain immediately relevant results on walking capacity and energy cost, because these subjects were accustomed to the new composite AFO for only 2 weeks before the measurements. Thijssen et al. (38) reported a greater improvement in energy cost after 3 weeks of familiarization with an AFO than an immediate effect. In another study, Erel et al. reported that the physiological cost index decreased significantly after 3 months of using a dynamic AFO (39). These interesting and immediate effects suggest that the potential benefits of a C-AFO on energy cost should be evaluated after a longer period of use and probably in subacute poststroke patients to assess its long-term efficacy.

Although both AFOs seem to provide benefits, some particularities such as the presence of knee hyperextension or flexed knee should be considered before recommending a particular type of AFO. Indeed, although our results must be interpreted with caution due to our small sample size, they suggest that, in patients with knee hyperextension, standard and ready-to-wear composite AFOs can be a good alternative to custom-made AFOs, as walking speed with the C-AFO is faster than with the P-AFO. Nevertheless, in addition to the question of the AFO composite, we can also ask whether an anterior AFO might not be better for patients with stroke with a flexed-knee gait.

### Conclusion

The use of a new off-the-shelf composite AFO in patients with chronic stroke may immediately increase walking speed, improve gait variables, and decrease the energy cost of walking as effectively as the patient’s usual plastic custom-made AFO. The functional adaptation of the patients to the C-AFO seems relatively fast and efficient since the habituation period was only 2 weeks, and the reported satisfaction was high.
